# Body mass composition analysis as a predictor of overweight and obesity in children and adolescents

**DOI:** 10.3389/fpubh.2024.1371420

**Published:** 2024-04-24

**Authors:** Bartosz Aniśko, Idzi Siatkowski, Małgorzata Wójcik

**Affiliations:** ^1^Department of Physiotherapy, Faculty of Sport Sciences in Gorzów Wielkopolski, Poznań University of Physical Education, Gorzów Wielkopolski, Poland; ^2^Department of Mathematical and Statistical Methods, Poznan University of Life Science, Poznań, Poland

**Keywords:** body composition analysis, children’s health, overweight and obesity factors, metabolic risk, body weight of growing children

## Abstract

**Introduction:**

Body mass composition is directly related to health and its disorders are correlated with diseases such as obesity, diabetes, osteoporosis and sarcopenia. The purpose of this study was to analyze body mass composition among traditional elementary school students and ballet school students.

**Methods:**

A total of 340 students participated in the study, 95 of whom attended ballet school and 245 elementary school students. A Tanita BC-418 MA analyzer was used to analyze body mass composition. Such body composition indices as BMI (Body Mass Index), muscle mass, fat mass, lean body mass and water content were evaluated.

**Results:**

The results show statistical significance for BMI between high school ballet students and elementary school ballet students, as well between high school ballet students and elementary school students. Comparisons in relation to gender and schools BMI, statistical significance was obtained for: BHSw (ballet high school women) and EBSw (elementary ballet school women), BHSw and ESw (elementary school women), BHSm (ballet high school men) and EBSm (elementary ballet school men), and between BHSm and ESw. Comparing muscle mass index (kg) between ballet high school and elementary school, between ballet high school and elementary school, and between ballet high school and elementary school - statistical significance was obtained for all comparisons. Analyzing in pairwise comparisons by gender and school achieved a statistically significant difference for: BHSw and EBSw, BHSm and EBSw, EBSw and ESm. Comparing the fat mass index (kg), no significant differences were observed between the analyzed schools and the school and gender of the children studied. The value of the lean body mass index differed significantly between groups by school and gender. Comparing the water content index, statistically significant differences were obtained for school and gender.

**Discussion:**

The body mass composition of ballet school students differs from that of standard school students.

## 1 Introduction

The composition of human body mass informs about the content of the various components that make up the human body. The basic components that make up human body mass include fat tissue, muscle tissue, skeletal system, organ mass, and water (1). Adequate proportions of the above-mentioned components determine a person’s health and nutritional status. Also in children, despite differentiated and dynamic growth, indicators in the composition of body mass can be a predictor of future metabolic problems (2, 3). One of the key components of body composition is total water content. In a healthy person, the ratio of total water content should be 60% in men and 50% in women. In addition to being an indication of proper hydration, this parameter is also important information, for example, when dosing drugs (4). Another component mentioned is lean body mass. Fat-free body mass consists of water content, muscle mass and bone mass (5). Adipose tissue in od-responding amounts is an equally important component of body composition, but its pathological accumulation is intrinsically linked to obesity (6). Studies show that the ratio of body fat to lean body mass is one of the predictors of premature death (7). The ratio of body fat to lean body mass can also be used as one of the diagnostic factors in obesity (8). Muscle mass is an essential element for staying healthy and independent. In the aging process, we lose muscle mass and by the age of 80 we can lose up to 30% of it, and one of the main reasons for this is lack of physical activity (9). Abnormal body mass composition is associated with cardiovascular disease, cancer, diabetes or osteoporosis (10, 11). Detailed analysis of body mass composition can serve as a diagnostic method in disease entities such as obesity or sarcopenia (12) and can be a basic screening test among children and adolescents and detect abnormal weight problems at an early stage. One of the basic tools for universal weight assessment is the Body Mass Index (BMI). It is a ratio formed by dividing body weight given in kilograms by the square of height given in meters (13). The value of the BMI index allows a preliminary assessment of the nutritional status of the examinee and classify him into the appropriate group. The basic states of body mass possible to diagnose with the help of BMI are: underweight (<18.5 kg/m^2^), overweight (25.0–29.9 kg/m^2^), obesity (>30 kg/m^2^) and normal weight (18.5–24.9 kg/m^2^) (14). The trend analysis used World Health Organization (WHO) data extracted from the latest (fifth) phase of the European Childhood Obesity Surveillance Initiative (15), conducted between 2018 and 2020. The information was provided by 33 countries. The total number of children surveyed was nearly 411,000. The data collected provides information that 29% of children aged 7–9 are living with overweight or obesity (15). Projections of global rates of overweight and obesity by the World Obesity Federation (WOF) suggest that more than 4 billion individuals could be affected by 2035, compared to more than 2.6 billion in 2020. The prevalence of obesity alone is projected to increase from 14 to 24% of the population over the same period, affecting nearly 2 billion adults, children and adolescents by 2035. The trend of increasing obesity prevalence is expected to be particularly noticeable among children and adolescents, predicting an increase in the percentage of boys from 10 to 20% globally between 2020 and 2035, and a similar increase in the percentage of girls from 8 to 18% globally (16). The overarching goal in the treatment of overweight and obesity is controlled loss of excess body weight. Childhood obesity carries serious health consequences such as hypertension, type 2 diabetes, dyslipidemia, post-natal complications, polycystic ovary syndrome, respiratory dysfunction, musculoskeletal impact, renal complications, neurological complications, and overall impact on adolescence (17). However, it is important to note that underweight is an equally serious problem, as the WHO reports that more than 462 million people worldwide are affected by this problem. In 2016, being underweight contributed to one million deaths worldwide (18). Treatment of obesity should be comprehensive and may consist of factors such as lifestyle changes, cooperation with a nutritionist, pharmacotherapy and in some cases even surgical intervention (19). There are still no drugs with high efficacy in the treatment of obesity, but new ones are constantly being developed that may revolutionize the approach to this disease in the future (20). Bariatric surgery is an effective solution to reduce the risk of death from obesity and its consequences, but it is a highly invasive method and is often a last resort (19). Physical activity is primarily a preventive measure in maintaining health and normal body weight, but also modulating its level in overweight and obese people can effectively increase energy expenditure and consequently lead to the restoration of the expected body weight. Appropriate physical activity is able to improve respiratory, cardiovascular or other metabolic functions which, combined with the loss of excess body weight, will contribute to a significant improvement in overall health (21). The problem of weight disorders among children and adolescents is a global problem, and there is a need for this phenomenon to be explained in depth and precision. We undertook this topic to provide detailed information on the body composition of children of different ages and to find correlations in the various components in relation to overweight and obesity. We decided to include children practicing ballet in the study due to the fact that it is a demanding discipline that forces a high level of physical activity and fitness.

Considering the complexity of the problem, which is overweight and obesity among children and adolescents, the following research hypotheses were established:

1. Body mass index (BMI) values and body mass composition differ between elementary school students and ballet school students.
2. The composition of body weight differs between the sexes and ballet school students will be characterized by a higher incidence of normal body weight.

## 2 Materials and methods

### 2.1 Study group

**Figure 1:**
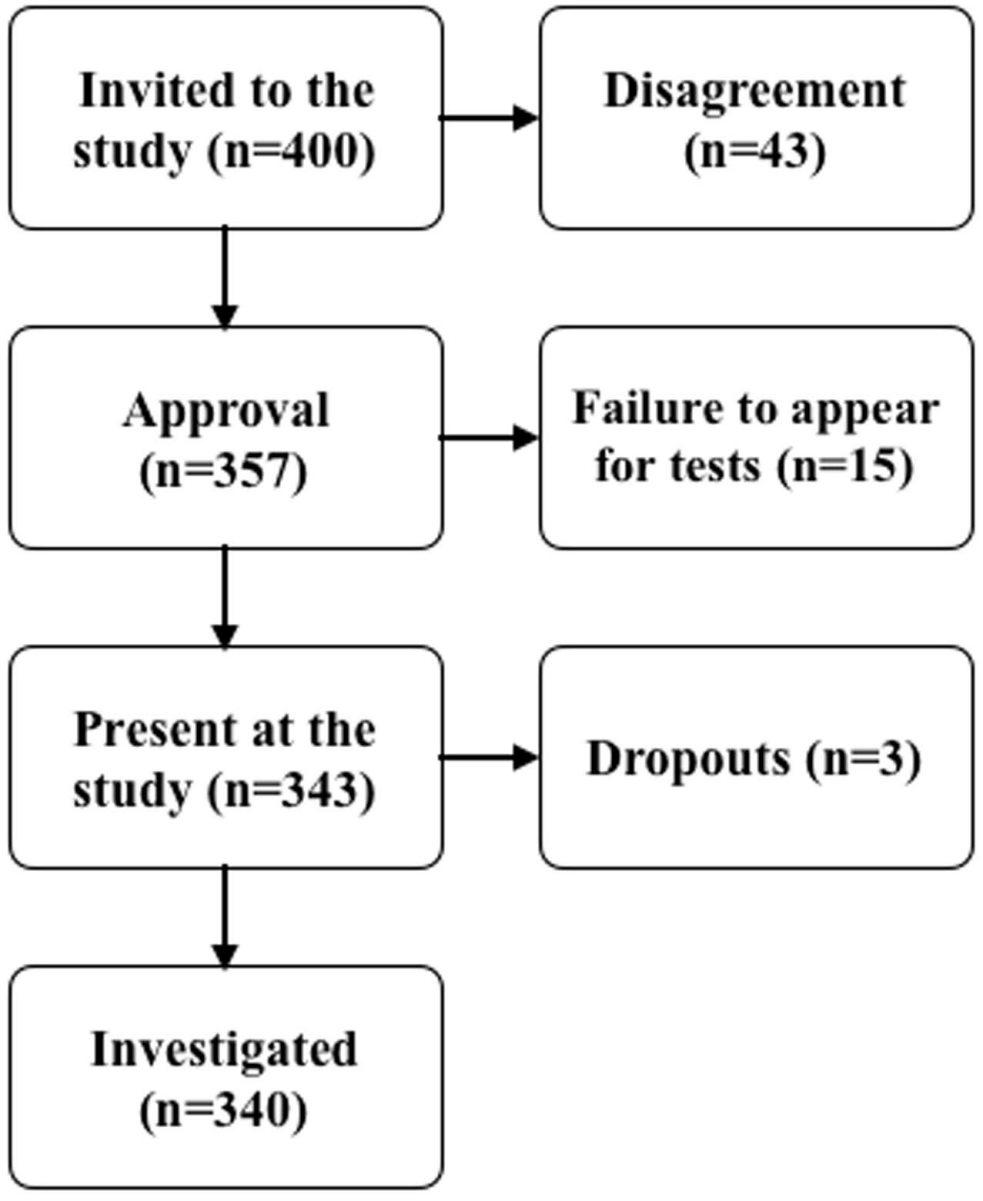
Flowchart showing the recruitment of participants.

The research was organized in cooperation with elementary schools and high schools. All students from these schools were invited to participate in the study. The study invited 400 participants. 43 (11%) of those invited did not agree to participate in the study, 15 (4%) were absent on the days of the study and 3 (1%) of the results were rejected (Figure 1). A total of 340 students participated in the study, 95 of whom attended ballet school (including 85 girls and 10 boys, aged 14 ± 2) and 245 elementary school students (regular school, including 122 girls and 123 boys, aged 10 ± 2) (Figure 1). In the analysis, students were divided into groups ballet high school (BHS), elementary ballet school (EHS) and elementary school (ES). The study was based on ethical guidelines (22) and the approval of the bioethics committee resolution no. 559/23 dated June 29, 2023 and participants could enter the study after providing written consent from a parent or guardian.

### 2.2 Inclusion and exclusion criteria

Inclusion criterion: good health - no diseases of the circulatory, respiratory, digestive, nervous systems. No limitations of the musculoskeletal system that prevent standing, jumping, moving at a fast pace. Participants should be within the assumed age range of 6–18 years. Written consent of parents or guardians for participation of children and adolescents in the study. Exclusion criterion - no consent to participate in the study. The occurrence of diseases that disqualify from the study. Severe limitations of the musculoskeletal system preventing performance of tasks during the study.

### 2.3 Measurement of body mass composition

The study was performed using a Tanita BC-418 MA body composition analyzer (23). The apparatus used uses a non-invasive electrical bioimpedance method. This method is based on the measurement of the total electrical resistance of the body, which is the result of resistance (passive resistance) and reactance (active resistance). This measurement is carried out using a set of surface electrodes that are connected to a computer analyzer. The process uses a current of a certain frequency and intensity. The study was conducted in the morning (8:00–12:00) to reduce the risk of physical activity affecting the instrument reading. Participants entered the study dressed in light and comfortable sportswear. The test subject’s task was to step barefoot onto the testing apparatus (sensors under the feet) and grasp special handles (sensors at the hands), during the measurement the test subject stood upright looking ahead.

### 2.4 Percentile charts

In order to accurately interpret body mass index (BMI), centile grids created by the OLA and OLAF project, which was conducted in Poland between 2007 and 2013, were used. The centile grid is a statistical tool used in medicine and scientific research to analyze growth patterns, body weight and other developmental indicators of children and adolescents. It is a type of chart or table that shows the distribution of population data according to age and gender. The centile grid helps to assess whether a person’s development is in line with population norms (24, 25).

### 2.5 Statistical analysis

The analysis carefully examined various components of body composition, such as body weight (in kilograms), BMI (body mass divided by the square of height), body fat content (in kilograms), fat-free mass (in kilograms), muscle mass (in kilograms) and water content (in kilograms). Once the data was collected, statistical analysis was undertaken using the R package (26). It was verified whether there is a normal distribution using the Shapiro–Wilk normality test. The acquired data do not follow a normal distribution. Statistical methods and tests were used: descriptive statistics, Shapiro–Wilk test, Kruskal-Wallis test, Dunn test with adjust method Bonferroni. Dunn Test with adjust method Bonferroni was used for detailed comparison of BMI, muscle mass, fat mass and water index between schools. The Kruskal-Wallis test and Dunn test were used for multiple comparisons to compare BMI, muscle mass, fat mass and water indexes for students forming groups by school and gender. The results were presented using tables and graphs.

## 3 Results

First, descriptive statistics of BMI, water (kg), muscle mass (kg), fat mass (kg) and lean body mass (kg) were determined for ballet school (elementary and high school) and elementary school (Table 1). The next step in the statistical analysis was to use the Dunn test with the Bonferroni adjust method to compare each of the body mass composition indices between schools in detail. A check was then made with the Shapiro–Wilk Normality test to see if the data were subject to a normal distribution. The next step in the analysis was to compare body mass composition indices for students forming groups by school and gender. Basic scoring statistics were determined (Table 2), the assumption of normality of the data was checked, and the Kruskal-Wallis test and Dunn’s test of multiple comparisons were applied. Next, it was checked whether the conditions of normal distribution were met for the analyzed indicators by gender. Since the conditions of normal distribution were not met, the Kruskal-Wallis test was applied. Pairwise comparisons were then made for all indicators in relation to gender and schools (Tables 3, 4).

**Table 1:** Descriptive statistics of BMI (body mass index), TBW (total body water), LBM (lean body mass), FM (fat mass) MM (muscle mass) students of elementary school, ballet elementary school and ballet high school.

Index	Group	*n*	min	max	Median	iqr	mean	sd	se	ci
BMI	BHS	27	15.5	22.7	19.2	2.25	19.0	1.84	0.353	0.727
	EBS	68	13.2	23.2	17.0	2.52	17.5	2.03	0.246	0.491
	ES	245	12.5	38.3	17.1	4.80	18.5	4.42	0.282	0.556
TBW	BHS	27	29.6	56.0	40.7	3.20	41.4	5.99	1.150	2.370
	EBS	68	21.5	45.1	33.3	9.22	32.2	5.39	0.654	1.310
	ES	245	10.8	48.0	20.6	8.90	22.3	7.22	0.461	0.909
LBM	BHS	27	31.2	58.6	42.9	3.55	43.5	6.23	1.200	2.460
	EBS	68	22.4	47.5	35.0	9.60	33.8	5.67	0.688	1.370
	ES	245	14.7	65.6	28.2	12.2	30.5	9.86	0.630	1.240
FM	BHS	27	5.5	17.2	9.6	3.35	9.8	2.96	0.570	1.170
	EBS	68	4.6	17.3	8.5	3.02	8.9	2.93	0.355	0.709
	ES	245	2.8	46.3	7.7	5.70	9.9	6.79	0.434	0.855
MM	BHS	27	22.8	42.9	31.4	2.55	31.9	4.57	0.879	1.810
	EBS	68	16.4	34.8	25.6	7.00	24.8	4.15	0.503	1.000
	ES	245	14.1	62.6	27.0	11.3	29.2	9.36	0.598	1.180

BHS, ballet high school; EBS, elementary ballet school; ES, elementary school; min, minimum; max, maximum; iqr, interquartile range; sd, standard deviation; se, standard error; ci, confidence interval.

**Table 2:** Descriptive statistics for BMI by gender in schools.

Index	Group	*n*	min	max	median	iqr	Mean	sd	se	ci
BMI	BHSw	23	15.5	22.4	19.1	2.25	18.8	1.80	0.375	0.78
	BHSm	4	19.2	22.7	19.8	1.25	20.4	1.58	0.788	2.51
	EBSw	62	13.2	23.2	17.0	2.65	17.4	2.08	0.265	0.53
	EBSm	6	16.5	20.2	17.4	1.48	17.8	1.38	0.564	1.45
	ESw	122	12.6	32.2	17.0	4.18	18.0	3.96	0.358	0.71
	ESm	123	12.5	38.3	17.5	5.10	19.0	4.80	0.433	0.86
TBW	BHSw	23	29.6	48.2	40.5	2.60	39.6	4.14	0.864	1.79
	BHSm	4	46.8	56.0	52.0	6.28	51.7	4.35	2.170	6.92
	EBSw	62	21.5	45.1	32.8	8.82	31.7	5.35	0.679	1.36
	EBSm	6	34.6	40.9	36.8	3.32	37.4	2.45	1.000	2.57
	ESw	122	10.8	40.6	20.5	8.18	21.3	5.92	0.536	1.06
	ESm	123	12.2	48.0	21.4	11.4	23.4	8.20	0.739	1.46
MM	BHSw	23	22.8	37.3	31.3	2.00	30.5	3.22	0.672	1.40
	BHSm	4	35.8	42.9	39.8	4.85	39.6	3.36	1.680	5.35
	EBSw	62	16.4	34.8	25.2	6.88	24.4	4.14	0.526	1.05
	EBSm	6	26.4	31.3	28.0	2.55	28.5	1.90	0.777	2.00
	ESw	122	14.1	52.8	26.6	10.6	27.6	7.64	0.692	1.37
	ESm	123	16.1	62.6	28.0	14.8	30.7	10.6	0.956	1.89
FM	BHSw	23	5.5	17.2	9.7	2.80	10.1	3.00	0.626	1.30
	BHSm	4	6.4	10.9	7.2	2.17	7.92	2.10	1.050	3.34
	EBSw	62	4.6	17.3	8.8	2.78	9.08	2.94	0.373	0.75
	EBSm	6	5.1	10.6	6.4	1.98	6.87	2.06	0.842	2.17
	ESw	122	3.3	35.5	8.0	5.78	9.87	5.77	0.523	1.03
	ESm	123	2.8	46.3	7.3	5.35	9.86	7.70	0.694	1.37
LBM	BHSw	23	31.2	50.9	42.7	2.75	41.7	4.39	0.915	1.90
	BHSm	4	48.9	58.6	54.4	6.55	54.1	4.57	2.280	7.27
	EBSw	62	22.4	47.5	34.4	9.43	33.4	5.65	0.718	1.44
	EBSm	6	36.0	42.8	38.2	3.48	39.0	2.63	1.070	2.76
	ESw	122	14.7	55.5	28.0	11.2	29.0	8.09	0.732	1.45
	ESm	123	16.6	65.6	29.2	15.6	32.0	11.2	1.010	2.00

Blk, ballet high school women; blm, ballet high school men; bpk, ballet elementary school men; bpk, ballet elementary school women; pk, elementary school women; pm, elementary school men; min, minimum; max, maximum; iqr, interquartile range; sd, standard deviation; se, standard error; ci, confidence interval.

**Table 3:** Comparison of BMI and TBW in relation to gender and schools.

Index	group1	group2	n1	n2	Statistic	*p*-value	Significance
BMI	BHSw	BHSm	23	4	0.958	0.338	ns
	BHSw	EBSw	23	62	−2.26	0.024	*
	BHSw	EBSm	23	6	−0.683	0.494	ns
	BHSw	ESw	23	122	−2.43	0.015	*
	BHSw	ESm	23	123	−1.50	0.135	ns
	BHSm	EBSw	4	62	−2.08	0.038	*
	BHSm	EBSm	4	6	−1.29	0.197	ns
	BHSm	ESw	4	122	−2.11	0.035	*
	BHSm	ESm	4	123	−1.69	0.091	ns
	EBSw	EBSm	62	6	0.558	0.577	ns
	EBSw	ESw	62	122	0.0018	0.999	ns
	EBSw	ESm	62	123	1.36	0.174	ns
	EBSm	ESw	6	122	−0.570	0.569	ns
	EBSm	ESm	6	123	−0.0639	0.949	ns
	ESw	ESm	122	123	1.66	0.098	ns
TBW	BHSw	BHSm	23	4	0.583	1.000	ns
	BHSw	EBSw	23	62	−2.60	0.139	ns
	BHSw	EBSm	23	6	−0.265	1.000	ns
	BHSw	ESw	23	122	−8.25	<0.0001	****
	BHSw	ESm	23	123	−7.31	<0.0001	****
	BHSm	EBSw	4	62	−1.84	0.978	ns
	BHSm	EBSm	4	6	−0.678	1.000	ns
	BHSm	ESw	4	122	−4.31	<0.001	***
	BHSm	ESm	4	123	−3.89	0.002	**
	EBSw	EBSm	62	6	1.20	1.000	ns
	EBSw	ESw	62	122	−7.95	<0.0001	****
	EBSw	ESm	62	123	−6.59	<0.0001	****
	EBSm	ESw	6	122	−4.19	<0.0001	***
	EBSm	ESm	6	123	−3.68	0.003	**
	ESw	ESm	122	123	1.67	1.000	ns

BHSw, ballet high school women; BHSm, ballet high school men; EBSm, elementary ballet school men; EBSw, elementary ballet school women; ESw, elementary school women; ESm, elementary school men; *p*, *p*-value; *p*.adj, adjusted *p*-value; *p*.adj.signif, adjusted *p*-value with significance; ns, not significant; *, significant codes; − ****, *p*-value <0.0001; ***, *p*-value <0.001; **, *p*-value <0.01; *, *p*-value <0.05.

**Table 4:** Comparison of MM and LBM in relation to gender and schools.

Index	Group1	group2	n1	n2	Statistic	*p*-value	Significance
MM	BHSw	BHSm	23	4	1.48	1.000	ns
	BHSw	EBSw	23	62	−4.11	<0.0001	***
	BHSw	EBSm	23	6	−0.61	1.000	ns
	BHSw	ESw	23	122	−2.63	0.130	ns
	BHSw	ESm	23	123	−1.65	1.000	ns
	BHSm	EBSw	4	62	−3.50	0.007	**
	BHSm	EBSm	4	6	−1.68	1.000	ns
	BHSm	ESw	4	122	−2.75	0.089	ns
	BHSm	ESm	4	123	−2.32	0.309	ns
	EBSw	EBSm	62	6	1.69	1.000	ns
	EBSw	ESw	62	122	2.60	0.138	ns
	EBSw	ESm	62	123	4.04	<0.0001	***
	EBSm	ESw	6	122	−0.76	1.000	ns
	EBSm	ESm	6	123	−0.22	1.000	ns
	ESw	ESm	122	123	1.74	1.000	ns
LBM	BHSw	BHSm	23	4	0.97	1.000	ns
	BHSw	EBSw	23	62	−3.58	0.005	**
	BHSw	EBSm	23	6	−0.47	1,000	ns
	BHSw	ESw	23	122	−6.31	<0.0001	****
	BHSw	ESm	23	123	−5.29	<0.0001	****
	BHSm	EBSw	4	62	−2.71	0.099	ns
	BHSm	EBSm	4	6	−1.15	1.000	ns
	BHSm	ESw	4	122	−3.86	0.002	**
	BHSm	ESm	4	123	−3.40	0.010	*
	EBSw	EBSm	62	6	1.54	1.000	ns
	EBSw	ESw	62	122	−3.59	0.005	**
	EBSw	ESm	62	123	−2.11	0.527	ns
	EBSm	ESw	6	122	−2.91	0.054	ns
	EBSm	ESm	6	123	−2.36	0.274	ns
	ESw	ESm	122	123	1.81	1,000	ns

BHSw, ballet high school women; BHSm, ballet high school men; EBSm, elementary ballet school men; EBSw, elementary ballet school women; ESw, elementary school women; ESm, elementary school men; *p*, *p*-value; *p*.adj, adjusted *p*-value; *p*.adj.signif, adjusted *p*-value with significance; ns, not significant; *, significant.

### 3.1 Body mass index (BMI)

**Figure 2:**
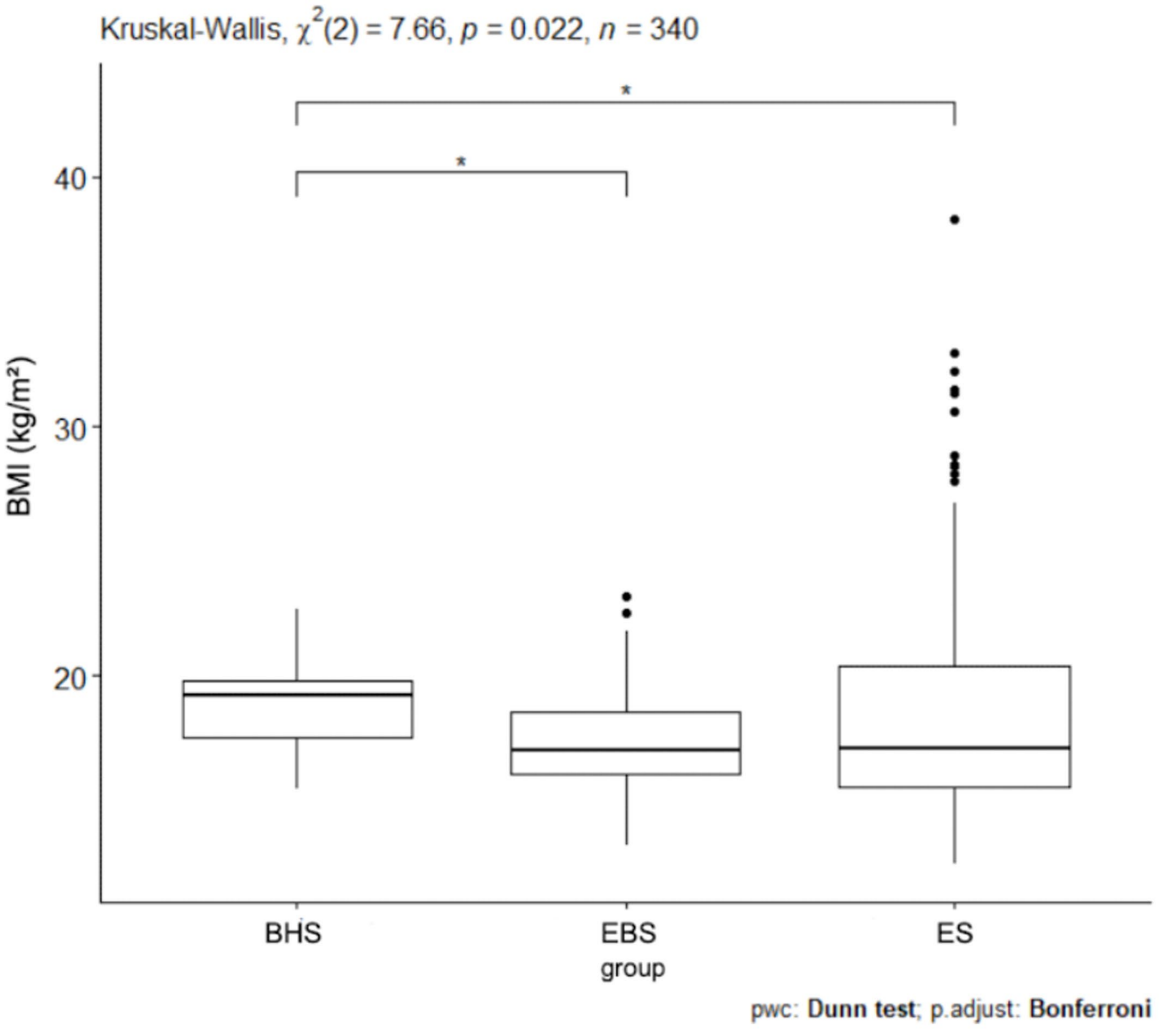
BMI values for ballet (elementary and high school) and regular elementary school students. BHS, ballet high school; EBS, elementary ballet school; ES, elementary school; BMI, body mass index.

**Figure 3:**
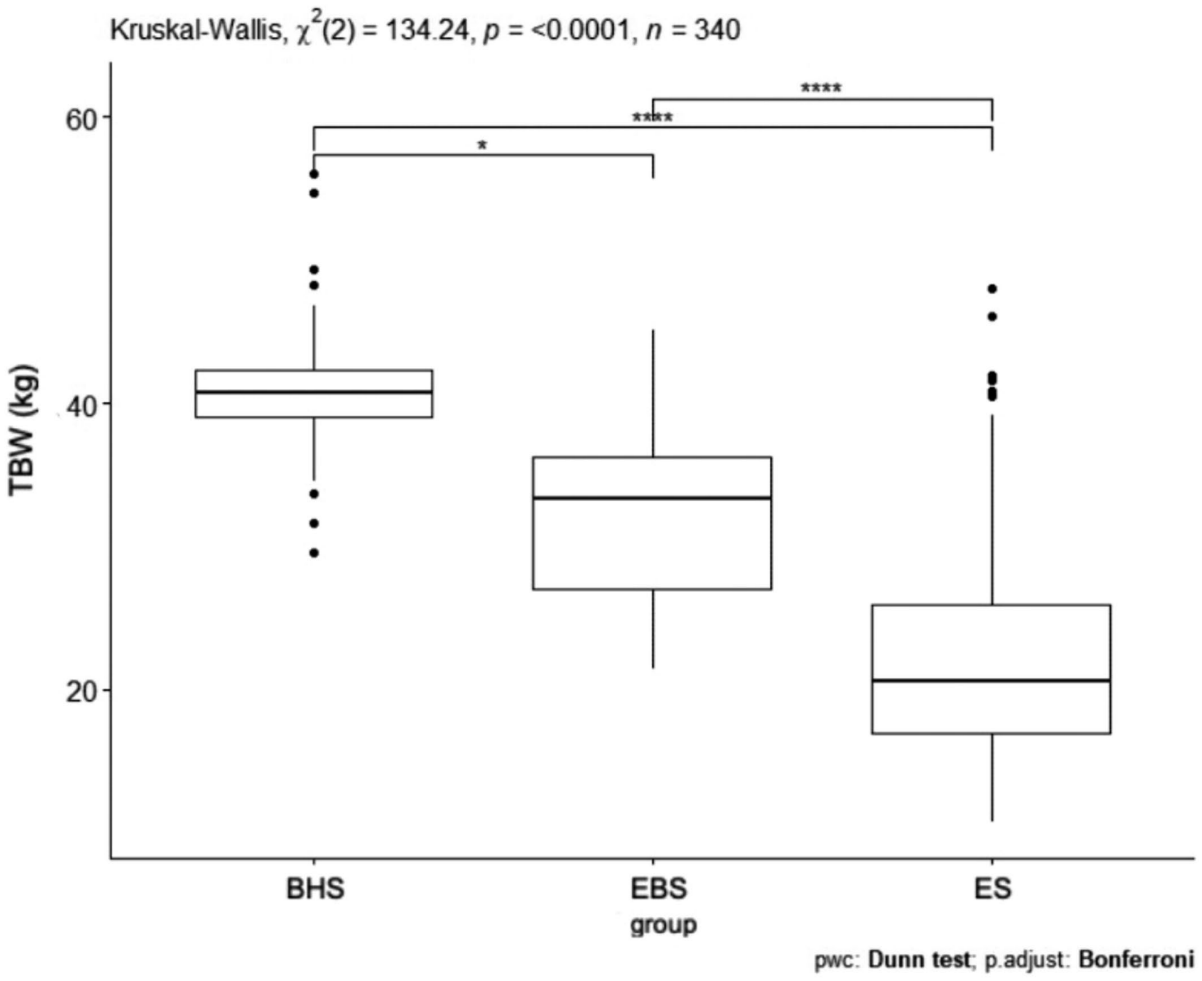
BMI values by sex in ballet and regular elementary school, BHSw, high school ballet women; BHSm, high school ballet men; EBSw, elementary ballet school women; EBSm, elementary ballet school men; ESw, elementary school women; ESm, elementary school men.

Among ballet school students (elementary and high school), 81% were of normal weight while 19% were underweight. No students were observed whose BMI indicated they were overweight or obese. In elementary school, 61% of students were characterized by normal weight, 20% were overweight, 7% were obese while 12% were underweight. Since normal distribution does not occur when comparing the BMI results of the three schools analyzed, the Kruskal-Wallis test was applied and *p* = 0.022 was obtained (Figure 2). The highest number of abnormal BMI results was observed in the elementary school. 3Statistical significance was obtained for BMI between high school ballet students and elementary school ballet students *p* = 0.0227 (Table 5), as well as statistical significance between high school ballet students and elementary school students *p* = 0.0301 (Table 5). In contrast, there is no statistical significance of BMI between sub-primary ballet school students and elementary school students. Analyzing BMI by gender, a *p* = 0.04 value was obtained (Figure 3). For pairwise comparisons in relation to gender and schools BMI, statistical significance was obtained for: BHSw and EBSw (*p* = 0.0238), BHSw and ESw (*p* = 0.0153), BHSm and EBSm (*p* = 0.0379), and between BHSm and ESw (*p* = 0.0351) (Table 3).

**Table 5:** Comparison of BMI, TBW, MM, FM, and LMB between schools.

Index	Group 1	group 2	n1	n2	Statistic	*p*-value	Significance
BMI	BHS	EBS	27	68	−2.67	0.023	*
	BHS	ES	27	245	−2.58	0.030	*
	EBS	ES	68	245	0.62	1.000	ns
TBW	BHS	EBS	27	68	−2.80	0.015	*
	BHS	ES	27	245	−8.95	<0.0001	****
	EBS	ES	68	245	−8.59	<0.0001	****
MM	BHS	EBS	27	68	−4.65	<0.0001	****
	BHS	ES	27	245	−2.98	0.009	**
	EBS	ES	68	245	3.31	0.003	**
FM	BHS	EBS	27	68	−1.27	0.612	ns
	BHS	ES	27	245	−2.10	0.107	ns
	EBS	ES	68	245	−1.00	0.952	ns
LBM	BHS	EBS	27	68	−3.93	<0.001	***
	BHS	ES	27	245	−6.88	<0.0001	****
	EBS	ES	68	245	−3.66	<0.001	***

BHS, ballet high school; EBS, elementary ballet school; ES, elementary school; *p*, *p*-value; *p*.adj, adjusted *p*-value; *p*.adj.signif, adjusted *p*-value with significance; ns, not significant, *, significant codes; − ****, *p*-value <0.0001, ***, *p*-value <0.001; **, *p*-value <0.01; *, *p*-value <0.05.

### 3.2 Muscle mass index

**Figure 4:**
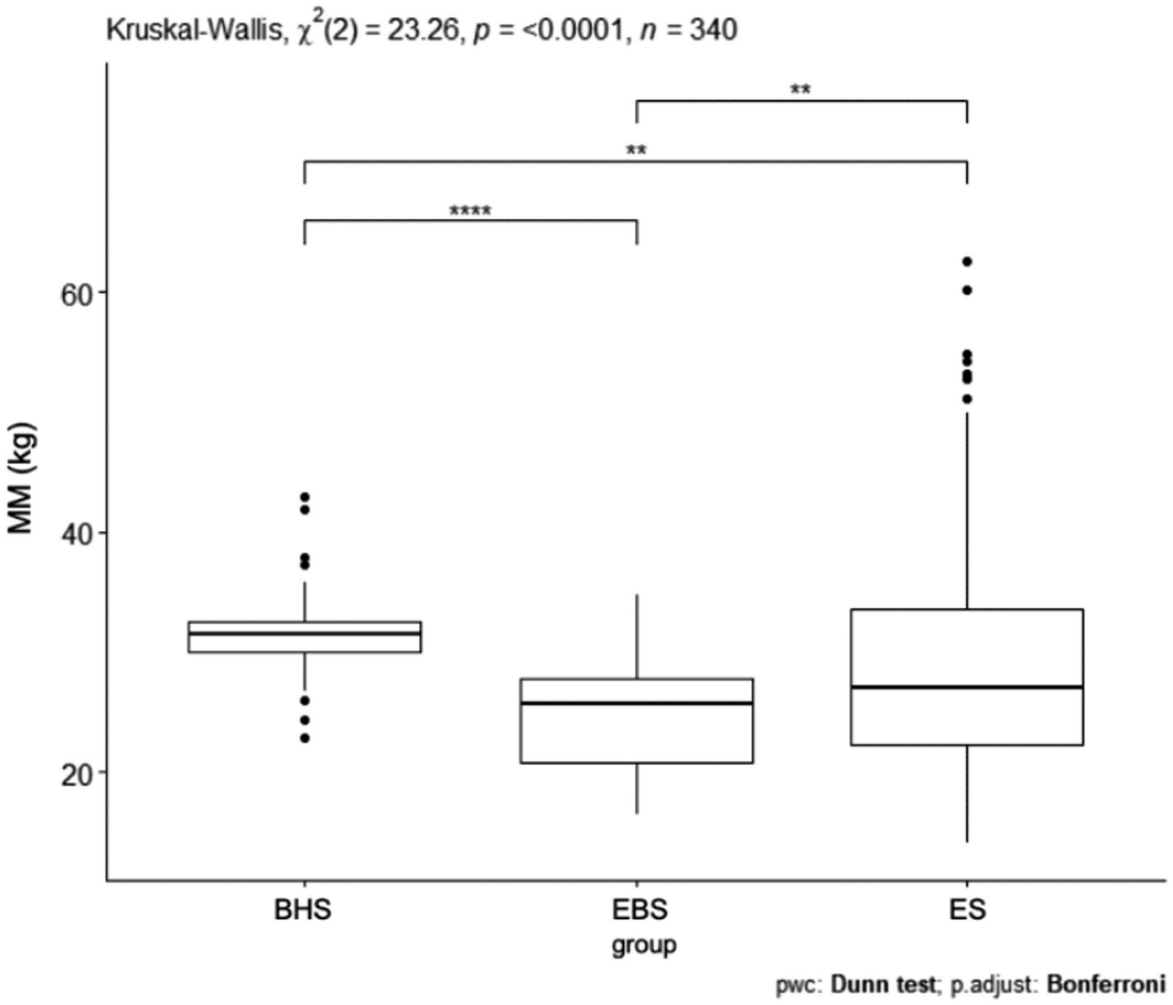
MM values for ballet (elementary and high school) and regular elementary school students. BHS, ballet high school; EBS, elementary ballet school; ES, elementary school; MM (muscle mass).

Comparing the muscle mass index, a *p* = 0.0001 was obtained (Figure 4). The schools were significantly different from each other and the largest difference was shown between ballet high school and ballet elementary school. In comparing the muscle mass index (kg) between all schools, statistical significance was obtained. Between ballet high school and elementary school *p* < 0.05, ballet high school and elementary school *p* = 0.009, ballet high school and elementary school *p* = 0.003 (Table 5). Analyzing the muscle mass index (kg) pairwise comparisons in relation to gender and schools, three combinations of groups achieved a statistically significant difference: BHSw and EBSw *p* = 0.001, BHSm and EBSw *p* = 0.007, EBSw and ESm *p* < 0.001 (Table 4).

### 3.3 Fat mass

**Figure 5:**
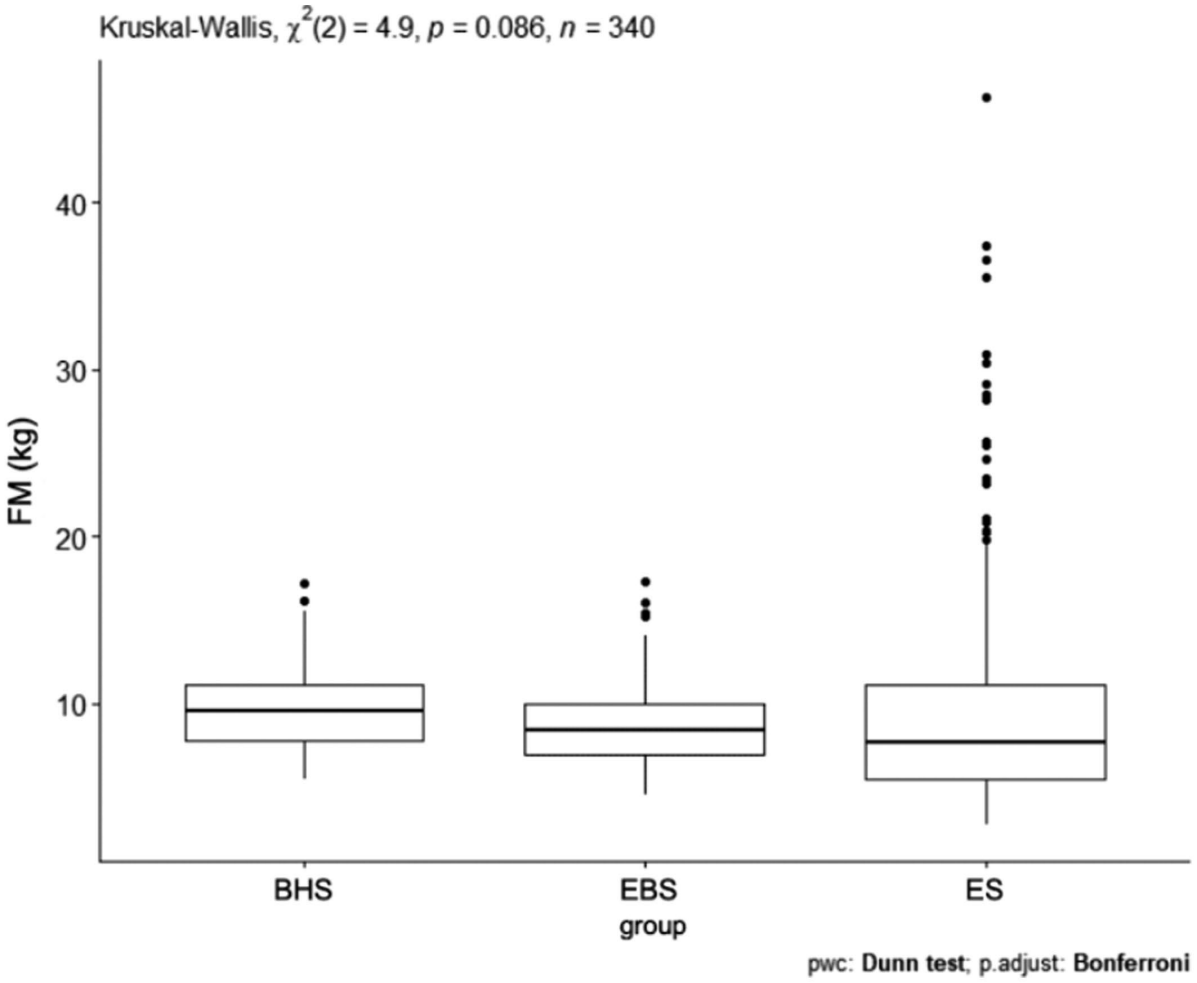
FM values for ballet (elementary and high school) and regular elementary school students. BHS, ballet high school; EBS, elementary ballet school; ES, elementary school; FM (fat mass).

**Figure 6:**
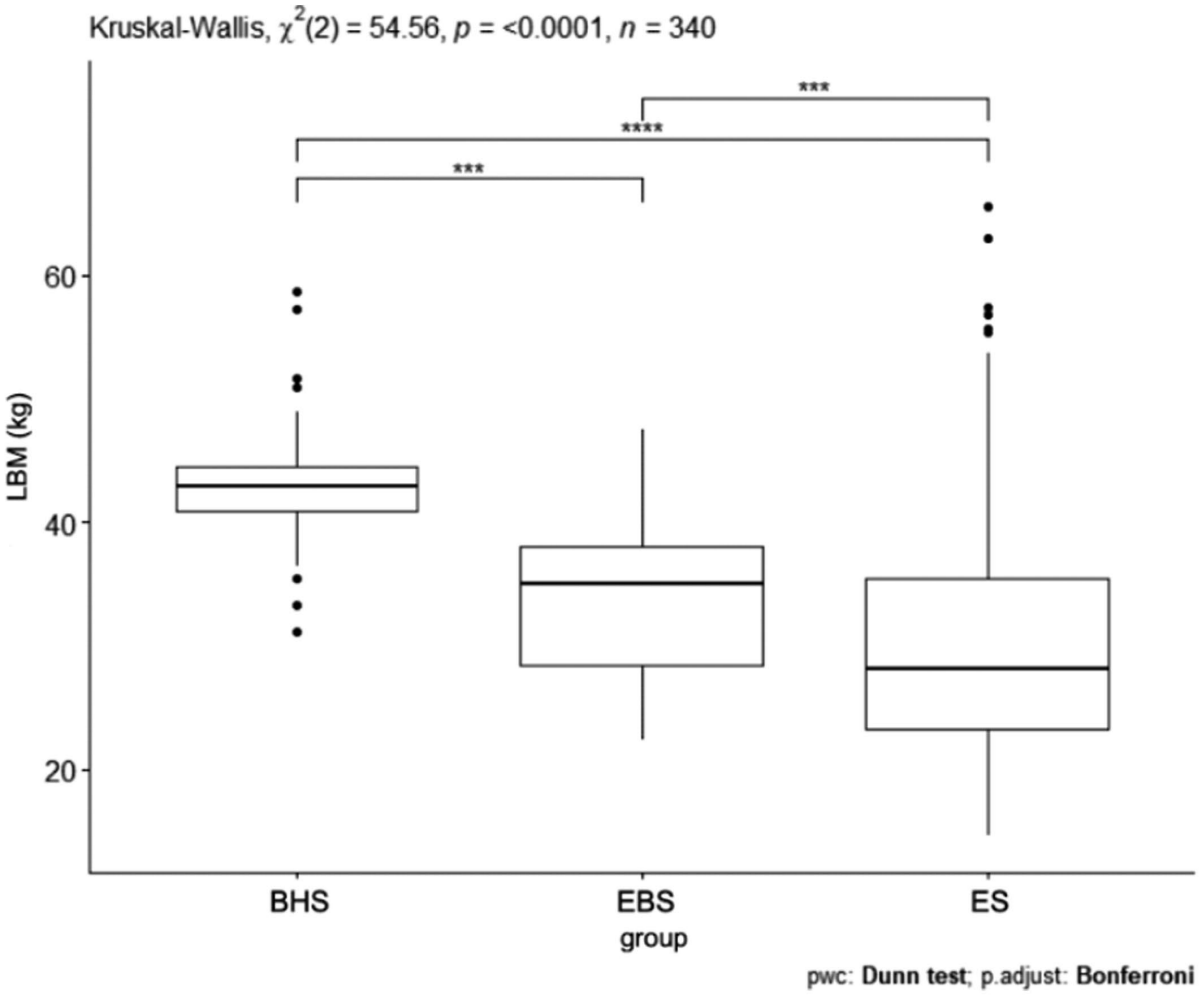
FM values by sex in ballet and regular elementary school; BHSw, high school ballet women; BHSm, high school ballet men; EBSw, elementary ballet school women; EBSm, elementary ballet school men; ESw, elementary school women; ESm, elementary school men.

Comparing the fat mass index, a *p* = 0.086 was obtained (Figure 5). The largest number of results significantly deviating in the fat mass level was recorded in the classical elementary school. Comparing the fat mass index (kg), no significant differences were observed between the analyzed schools (Table 5). There were no significant differences *p* = 0.052 in the comparison of fat mass considering the school and gender of the children studied (Figure 6). However, the most high scores in fat mass (kg) were observed in girls and boys from elementary school. In a comparison of fat mass index (kg), none of the group combinations achieved a difference at the level of statistical significance.

### 3.4 Lean body mass

**Figure 7:**
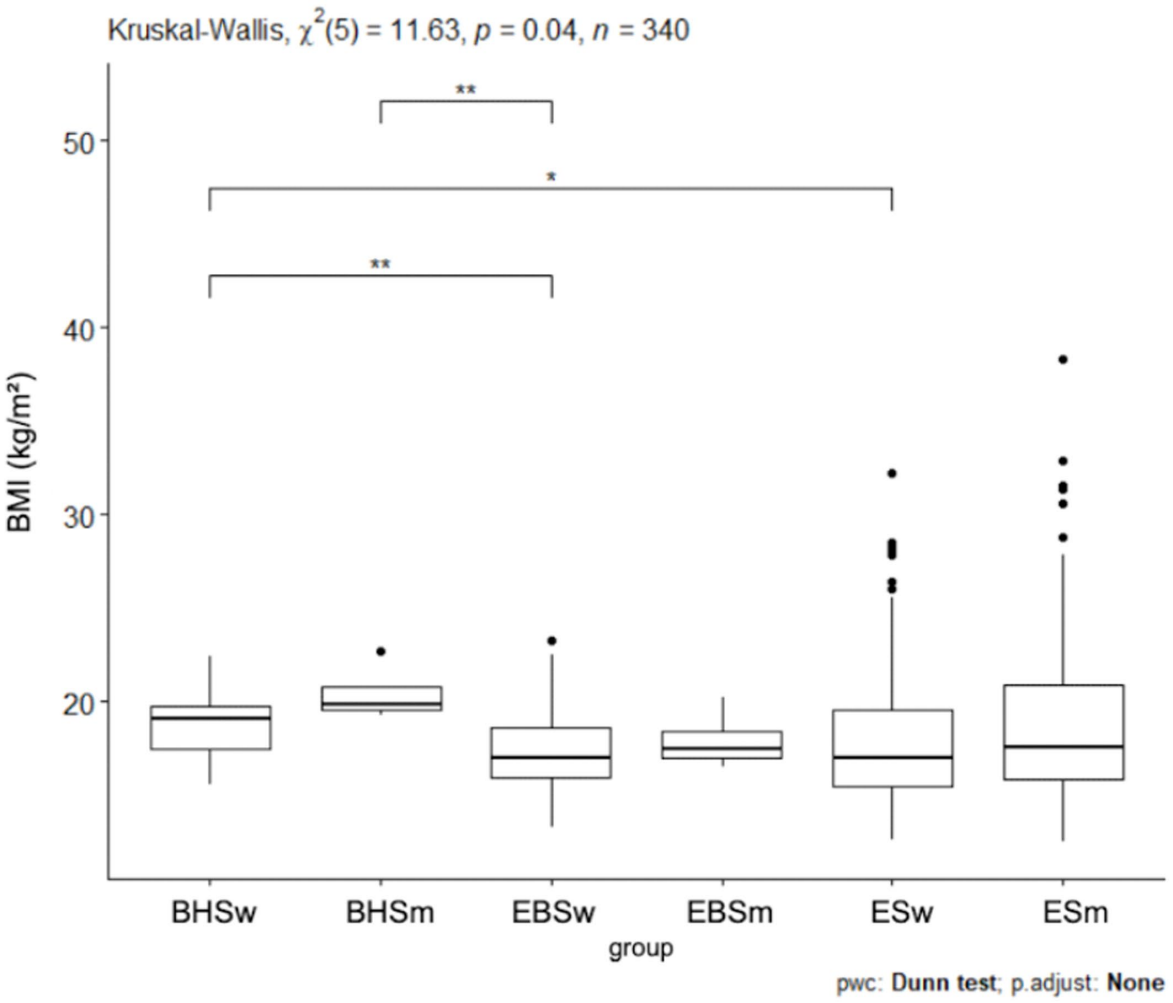
LBM values for ballet (elementary and high school) and regular elementary school students. BHS, ballet high school; EBS, elementary ballet school; ES, elementary school; LBM (lean body mass).

**Figure 8:**
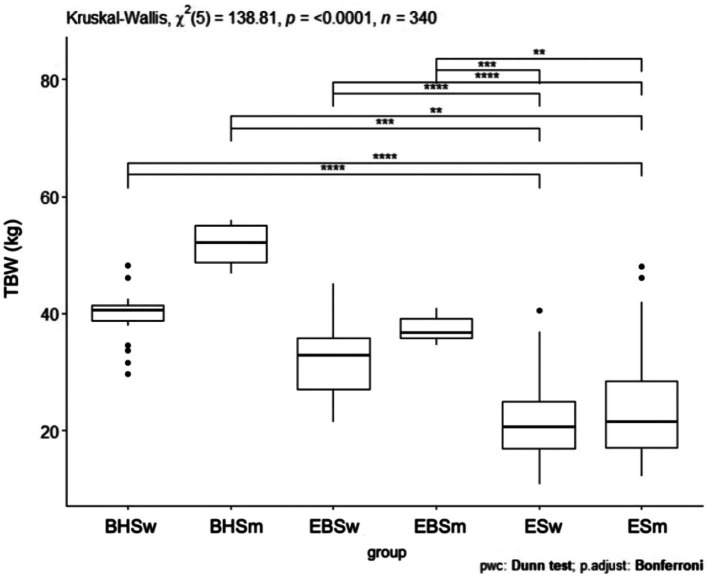
LBM values by sex in ballet and regular elementary school; BHSw, high school ballet women; BHSm, high school ballet men; EBSw, elementary ballet school women; EBSm, elementary ballet school men; ESw, elementary school women; ESm, elementary school men.

Comparing the lean body mass index, a *p* < 0.0001 was obtained (Figure 7). The schools were significantly different from each other and the largest difference was shown between ballet high school and ballet elementary school. Statistical significance was obtained in the comparison of lean body mass index (kg) between all schools. Between ballet high school and ballet elementary school *p* < 0.05, ballet high school and elementary school *p* < 0.05, ballet high school and elementary school *p* < 0.05 (Table 5). The value of the lean body mass index differed significantly between groups by school and gender *p* < 0.0001 (Figure 8). The largest difference was observed between ballet high school girls and elementary school girls and elementary school boys. The results of the lean body mass index (kg) show significant statistical differences in the following pairs of combinations: between the BHSw group and the ESw group, a *p*-value of *p* < 0.05 was obtained, between the BHSw group and the ESm group, a *p*-value of *p* < 0.05 was obtained, between the BHSm group and the ESw group, a *p*-value of *p* < 0.05 was obtained, between the BHSm group and the ESm group, a *p*-value of *p* < 0.05 was obtained, between the EBSw group and the ESw group, a *p*-value of *p* < 0.05 was obtained, between the EBSw group and the ESm group, a *p*-value of *p* < 0.05 was obtained, between the EBSm group and the ESw group, a *p*-value of *p* < 0.05 was obtained, between the EBSm group and the ESm group, a *p*-value of *p* < 0.05 was obtained (Table 4).

### 3.5 Total body water

**Figure 9:**
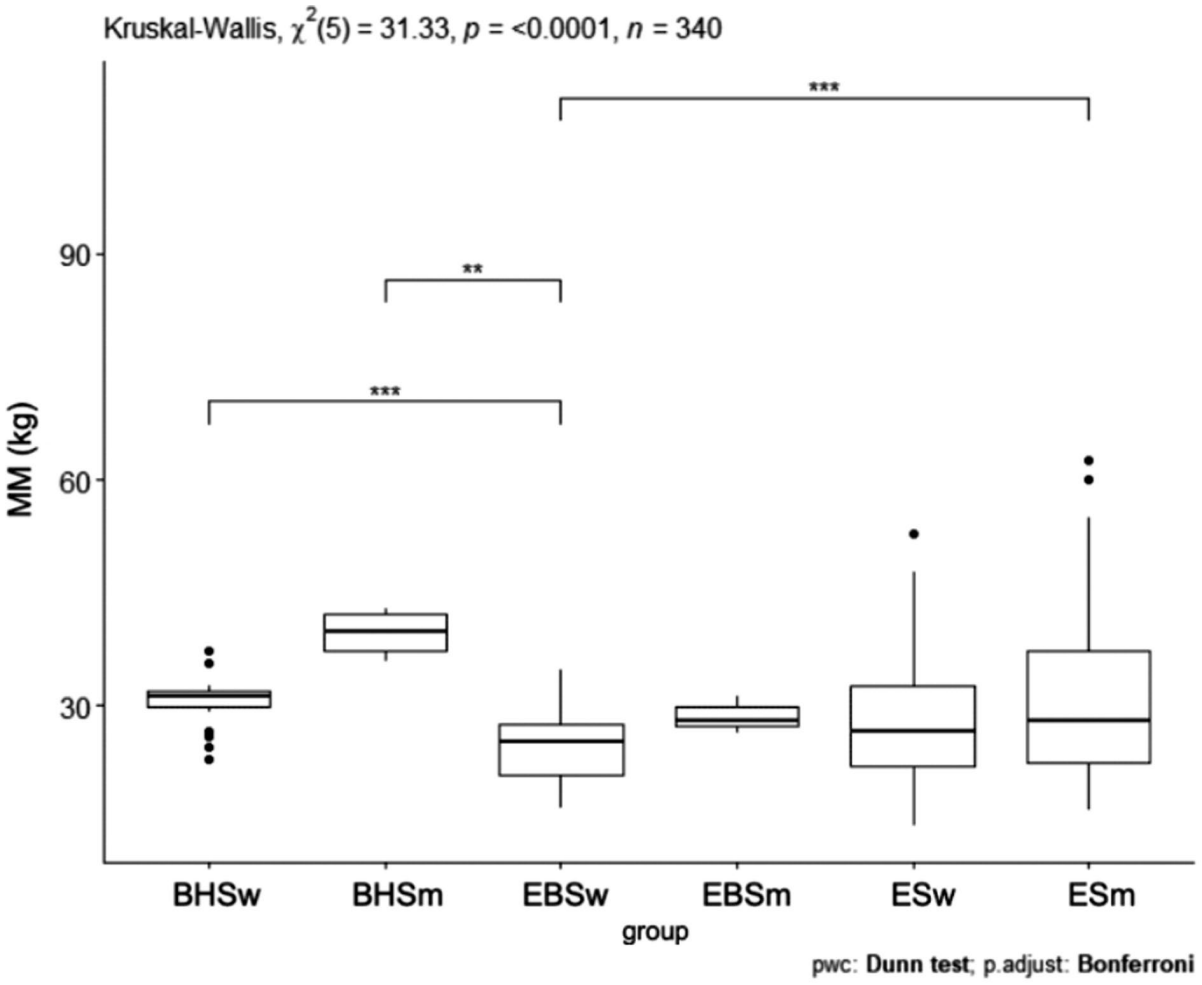
TBW values for ballet (elementary and high school) and regular elementary school students. BHS, ballet high school; EBS, elementary ballet school; ES, elementary school; MM (muscle mass).

**Figure 10:**
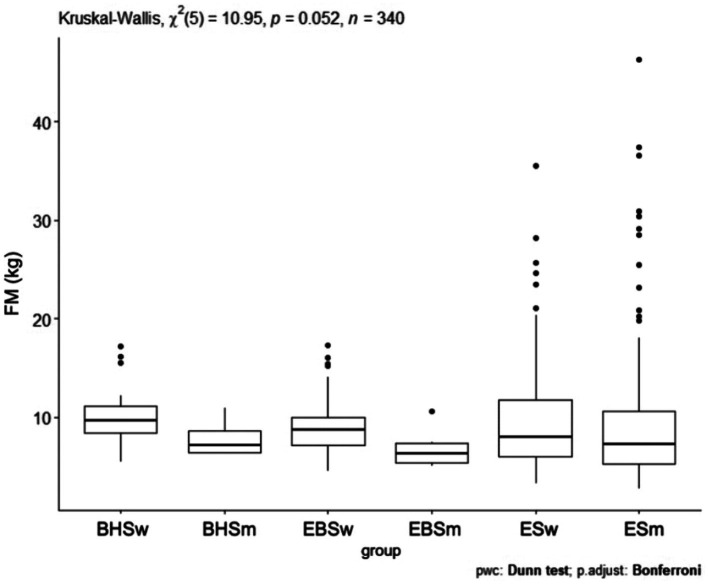
TBW values by sex in ballet and regular elementary school; BHSw, high school ballet women; BHSm, high school ballet men; EBSw, elementary ballet school women; EBSm, elementary ballet school men; ESw, elementary school women; ESm, elementary school men.

**Figure 11:**
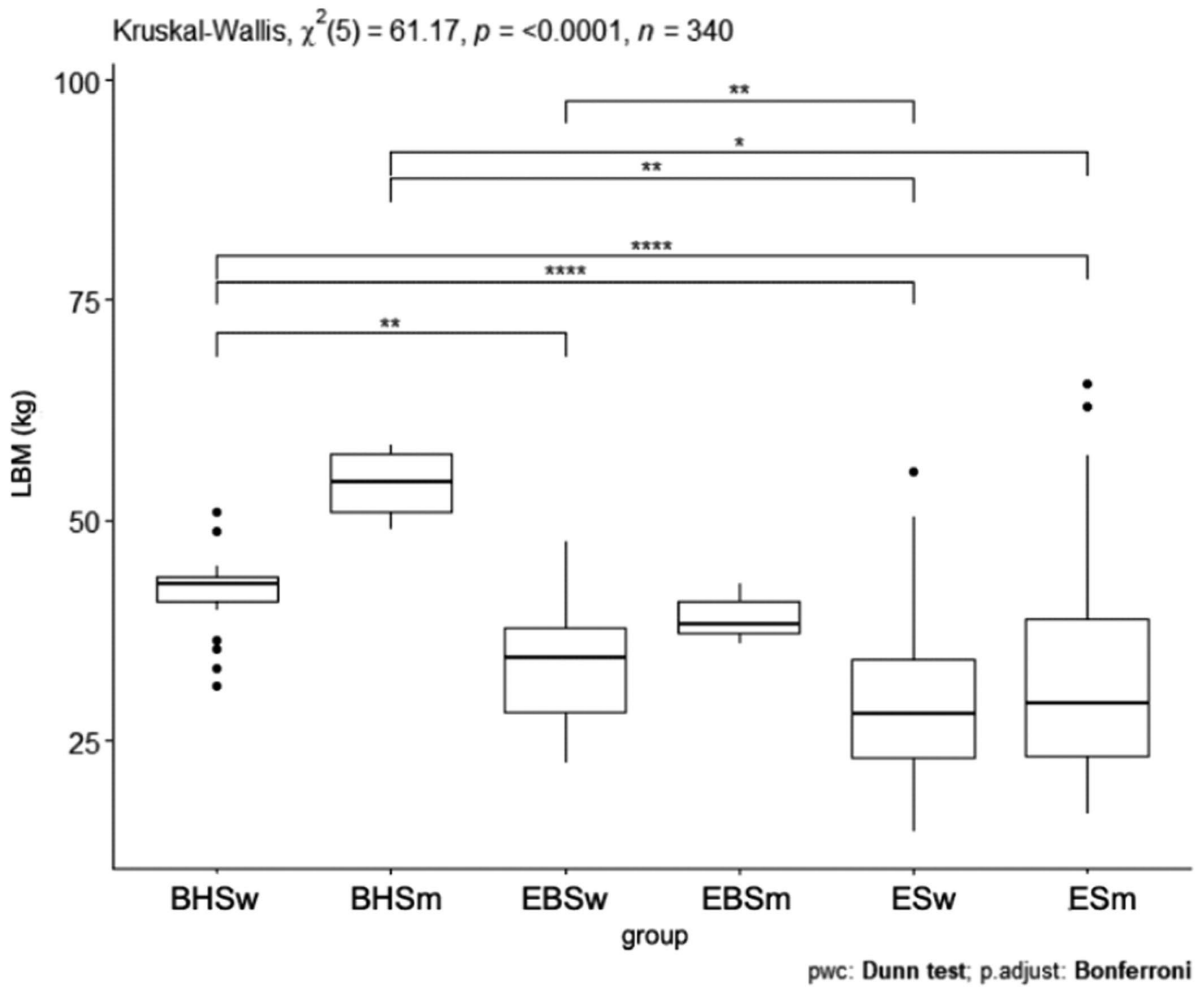
MM values by sex in ballet and regular elementary school; BHSw, high school ballet women; BHSm, high school ballet men; EBSw, elementary ballet school women; EBSm, elementary ballet school men; ESw, elementary school women; ESm, elementary school men.

Comparing the water content index, a *p* < 0.0001 was obtained (Figure 9). The schools were significantly different from each other and the largest difference was shown between the two ballet schools and the elementary school. In comparing the water content index (kg) between all schools, statistical significance was obtained. Between ballet high school and ballet elementary school *p* < 0.05, ballet high school and elementary school *p* < 0.05, ballet elementary school and elementary school *p* < 0.05 (Table 5). Comparing water content (kg) by school and gender, a *p* < 0.0001 was obtained (Figure 10). The biggest difference was observed between the group of girls and boys from the ballet high school and elementary school and the group of girls and boys from the primary school. Both boys and girls from the elementary school had the lowest body water content of all the groups studied. There was a significant difference between women from a ballet high school and women from a ballet elementary school, men from a ballet high school and men from a sub-primary ballet school, and between women from a primary ballet school and men from an elementary school *p* < 0.0001 (Figure 11). For the analyzed water quantity index (expressed in kilograms), statistical significance was obtained in the context of different combinations of variables: BHSw and ESw (*p* < 0.05), BHSw and ESm (*p* < 0.05), BHSm versus ESw (*p* < 0.05), BHSm and ESm (*p* < 0.05), EBSw and ESw (*p* < 0.05), EBSw and ESm (*p* < 0.05), EBSm and ESw (*p* < 0.05) and EBSm and ESm (*p* < 0.05) (Table 3).

## 4 Discussion

In the face of the pandemic of problems with excessive body weight, constant monitoring of children and adolescents with body composition analyzers is justified. The simplicity of testing with the analyzer with simultaneous broad diagnostic capabilities makes this type of testing an excellent screening tool and provides information about the health of the population.

The results obtained showed that the lowest BMI values were characterized by elementary ballet school students, followed by elementary school students and the highest by high school ballet students. Although the averaged BMI scores did not differ significantly from each other, significantly more scores above the acceptable healthy BMI level and indicative of overweight and obesity were observed in elementary school. Among ballet school students, not a single individual was observed characterized as overweight or obese, but 19% were underweight. The biggest difference was noted in the level of body water content. Children from both high school and elementary ballet school had significantly better hydration status compared to traditional elementary school. There were no significant differences in body fat among all the groups of children studied, but, as with BMI, the largest number of cases with increased body fat mass were found in elementary school. Both the index of muscle mass and lean body mass differed significantly in ballet high school boys compared to all other groups. However, these indices reportedly achieved levels among both ballet and traditional elementary school students. Ballet school students (elementary and high school) were more likely to have a more stable body mass composition compared to classical elementary school, which may indicate the positive impact of additional physical activity among students. It should also be noted that greater gender differences were observed in older children (high school) compared to elementary school-aged children.

The study by Karklin et al. examined children aged 9 and 10 years (*n* = 320). The results of this study show that 30% of the children studied were characterized by abnormal body weight. The authors point out that it was not only overweight and obesity that was a problem, but underweight, which was equally common (27). The study by Kobylinska et al. found similar results in terms of BMI in traditional elementary school students, with 57.5% of the subjects having normal body weight, 21.5% overweight, and 21% underweight. The study by Guzel et al. assessed children’s physical activity levels (*n* = 234 boys, 224 girls) and body mass composition. They found that higher levels of physical activity were associated with lower body fat mass in each age group regardless of gender which does not match the results obtained in our study (28). In the Mateo-Orcajada study, participants aged 12–16 years (*n* = 791) compared anthropometric characteristics including body composition of physically active and inactive individuals. As in our study, significant differences were noted in almost every indicator between the active and inactive groups (29). The study by Baran et al. evaluated children aged 6–17 years (*n* = 69). They found that higher levels of physical activity were associated with greater muscle mass and lean body mass among the study participants (30). Orntoft et al. studied 544 children aged 10–12 years by assessing their physical fitness and body mass composition. The subjects were divided into 4 activity groups: soccer, other “ball” sports, other sports or no physical activity. The body mass composition of the subjects did not differ statistically significantly between the groups. The group with the highest BMI, the highest body fat content, and the lowest muscle content was characterized by the group that did not play any sports (31). The study by Kazemi et al. included 150 female students (75 active and 75 non-active), in whom they also looked for differences in body mass composition. Statistically significant differences resulted in all body mass composition parameters assessed (32). In a similar study comparing the body mass composition of 337 female students by dividing them into active and inactive also a significant difference was observed in almost all body composition parameters. The difference was not observed only in lean body mass (kg), which is opposite to our work, where lean body mass was one of the most different parameters (33). A meta-analysis by Mateo-Orcajada clearly confirms the inconsistency of results regarding differences in body weight composition among children in different groups (34).

Given the great importance of normal body weight in childhood orkes on the incidence of weight problems in adulthood (35), the results presented here may significantly influence the recognition of the problem. The hypotheses we have put forward have been confirmed. The results presented will provide a better understanding of weight disorders among children and adolescents. In addition, they can be used by researchers and practitioners to plan programs to address weight problems.

## 5 Limitations

A limitation of the above work was that the group of students attending high school was too small and there was no comparison group of the same age. Another limitation was the lack of reference to other forms of physical activity, which translated into a lack of comparison between children and adolescents who play different sports. In addition, it would be a good idea to take measurements in preschool-aged children and repeat them periodically throughout adolescence.

## 6 Conclusion

All hypotheses were confirmed. BMI value and body mass composition differed between elementary school students and ballet school students. Body mass composition differed between genders and ballet school students were characterized by a higher prevalence of normal body mass. There is a lack of consistent evidence determining the body mass composition of specific groups in both children and adults, so further research is needed in this area.

## Data availability statement

The raw data supporting the conclusions of this article will be made available by the authors, without undue reservation.

## Ethics statement

The studies involving humans were approved by Komisja Bioetyczna przy Uniwersytecie Medycznym im. Karola Marcinkowskiego w Poznaniu ul. Bukowska 70, pok. A204 60-812 Poznań. The studies were conducted in accordance with the local legislation and institutional requirements. Written informed consent for participation in this study was provided by the participants' legal guardians/next of kin.

## Author contributions

BA: Conceptualization, Data curation, Formal analysis, Funding acquisition, Investigation, Methodology, Project administration, Resources, Software, Supervision, Validation, Visualization, Writing – original draft, Writing – review & editing. IS: Conceptualization, Data curation, Formal analysis, Funding acquisition, Investigation, Methodology, Project administration, Resources, Software, Supervision, Validation, Visualization, Writing – original draft, Writing – review & editing. MW: Conceptualization, Data curation, Formal analysis, Funding acquisition, Investigation, Methodology, Project administration, Resources, Software, Supervision, Validation, Visualization, Writing – original draft, Writing – review & editing.

## References

[1] Borga M West J Bell JD Harvey NC Romu T Heymsfield SB. Advanced body composition assessment: from body mass index to body composition profiling. J Investig Med. (2018) 66:1–9. doi: 10.1136/jim-2018-000722, PMID: 29581385 PMC5992366

[2] Weber DR Moore RH Leonard MB Zemel BS. Fat and lean BMI reference curves in children and adolescents and their utility in identifying excess adiposity compared with BMI and percentage body fat. Am J Clin Nutr. (2013) 98:49–56. doi: 10.3945/ajcn.112.053611, PMID: 23697708 PMC3683820

[3] Demerath EW Johnson W. Pediatric body composition references: what’s missing? Am J Clin Nutr. (2013) 98:1–3. doi: 10.3945/ajcn.113.064907, PMID: 23719556 PMC3683813

[4] Mattoo TK Lu H Ayers E Thomas R. Total body water by BIA in children and young adults with normal and excessive weight. PLoS One. (2020) 15:e0239212. doi: 10.1371/journal.pone.0239212 33031479 PMC7544096

[5] Merchant RA Seetharaman S Au L Wong MWK Wong BLL Tan LF. Relationship of fat mass index and fat free mass index with body mass index and association with function, cognition and sarcopenia in pre-frail older adults. Front Endocrinol. (2021) 12:765415. doi: 10.3389/fendo.2021.765415 PMC8741276 35002957

[6] Goossens GH. The metabolic phenotype in obesity: fat mass, body fat distribution, and adipose tissue function. Obes Facts. (2017) 10:207–15. doi: 10.1159/000471488, PMID: 28564650 PMC5644968

[7] Sedlmeier AM Baumeister SE Weber A Fischer B Thorand B Ittermann T. Relation of body fat mass and fat-free mass to total mortality: results from 7 prospective cohort studies. Am J Clin Nutr. (2021) 113:639–46. doi: 10.1093/ajcn/nqaa339 33437985

[8] Gazarova GM Bihari BM Soltis SJ. Fat and fat-free mass as important determinants of body composition assessment in relation to sarcopenic obesity. Rocz Panstw Zakl Hig. (2023) 74:59–69. doi: 10.32394/rpzh.2023.0243 37010407

[9] McLeod M Breen L Hamilton DL Philp A. Live strong and prosper: the importance of skeletal muscle strength for healthy ageing. Biogerontology. (2016) 17:497–510. doi: 10.1007/s10522-015-9631-7, PMID: 26791164 PMC4889643

[10] Holmes CJ Racette SB. The utility of body composition assessment in nutrition and clinical practice: an overview of current methodology. Nutrients. (2021) 13:2493. doi: 10.3390/nu13082493 34444653 PMC8399582

[11] Kuriyan R. Body composition techniques. Indian J Med Res. (2018) 148:648–58. doi: 10.4103/ijmr.IJMR_1777_18, PMID: 30666990 PMC6366261

[12] Sbrignadello S Göbl C Tura A. Bioelectrical impedance analysis for the assessment of body composition in sarcopenia and type 2 diabetes. Nutrients. (2022) 14:1864. doi: 10.3390/nu14091864, PMID: 35565832 PMC9099885

[13] Khanna D Peltzer C Kahar P Parmar MS. Body mass index (BMI): a screening tool analysis. Cureus. (2022) 14:e22119. doi: 10.7759/cureus.22119, PMID: 35308730 PMC8920809

[14] Zierle-Ghosh A Jan A. Physiology, body mass index. Treasure Island, FL: StatPearls Publishing (2023). 30571077

[15] WHO. Report on the fifth round of data collection, 2018–2020: WHO European childhood obesity surveillance initiative (COSI). (2022). Available at:https://www.who.int/europe/publications/i/item/WHO-EURO-2022-6594-46360-67071

[16] World Obesity Federation. (2023). World obesity atlas. Available at:https://www.worldobesityday.org/assets/downloads/World_Obesity_Atlas_2023_Report.pdf

[17] Medycyna Praktyczna Pediatria. Otyłość dziecięca Wydanie specjalne. (2023). Available at:https://www.mp.pl/nadwaga-i-otylosc/wytyczne/331397,otylosc-dziecieca

[18] Stanaway JD Afshin A Gakidou E. Global, regional, and national comparative risk assessment of 84 behavioral, environmental and occupational, and metabolic risks or clusters of risks for 195 countries and territories, 1990–2017: a systematic analysis for the global burden of disease study 2017. Lancet. (2018) 392:1923–94. doi: 10.1016/S0140-6736(18)32225-6, PMID: 30496105 PMC6227755

[19] Baker JS Supriya R Dutheil F Gao Y. Obesity: treatments, conceptualizations, and future directions for a growing problem. Biology. (2022) 11:160. doi: 10.3390/biology11020160, PMID: 35205027 PMC8869388

[20] Chakhtoura M Haber R Ghezzawi M Rhayem C Tcheroyan R Mantzoros CS. Pharmacotherapy of obesity: an update on the available medications and drugs under investigation. EClinicalMedicine. (2023) 58:101882. doi: 10.1016/j.eclinm.2023.101882 36992862 PMC10041469

[21] Pojednic R D’Arpino E Halliday I Bantham A. The benefits of physical activity for people with obesity, independent of weight loss: a systematic review. Int J Environ Res Public Health. (2022) 19:4981. doi: 10.3390/ijerph19094981, PMID: 35564376 PMC9102424

[22] Alderson P. Morrow V. (2011). The ethics of research with children and young people: a practical handbook. New York: SAGE Publications. 100–122.

[23] Thajer A Skacel G Truschner K Jorda A Vasek M Horsak B. Comparison of bioelectrical impedance-based methods on body composition in young patients with obesity. Children. (2021) 8:295. doi: 10.3390/children8040295, PMID: 33920492 PMC8070058

[24] Różdżyńska-Świątkowska A Kułaga Z Grajda A Gurzkowska B Góźdź M Wojtyło M. Wartości referencyjne wysokości, masy ciała i wskaźnika masy ciała dla oceny wzrastania i stanu odżywienia dzieci i młodzieży w wieku 3-18 lat. (2013). Available at:https://www.standardy.pl/artykuly/id/379

[25] Kułaga Z Różdżyńska-Świątkowska A Grajda A. Siatki centylowe dla oceny wzrastania i stanu odżywienia polskich dzieci i młodzieży od urodzenia do 18 roku życia. (2015). Available at:https://www.dziecizdrowoodzywione.pl/wp-content/uploads/2020/05/SIATKI-WHO-OLA-i-OLAF.pdf

[26] Hui EGM. Learn R for applied statistics: with data visualizations, regressions, and statistics. New York: Apress (2019).

[27] Karklina H Apinis P Kalnina L Sauka M Mozgis D Krumina D. Analysis of body composition of 9-and 10-year-old children in Latvia. Medicina (Kaunas). (2011) 47:573–8. doi: 10.3390/medicina47100082, PMID: 22186122

[28] Güzel Y Mustafa Atakan M Hüsrev Turnagöl H Nazan KŞ. Association of physical activity level with body composition in 12-14 years old children: a pilot study. Turk J Sports Med. (2022) 57:60–6. doi: 10.47447/tjsm.0616

[29] Mateo-Orcajada A Vaquero-Cristóbal R Esparza-Ros F Abenza-Cano L. Physical, psychological, and body composition differences between active and sedentary adolescents according to the “fat but fit” paradigm. Int J Environ Res Public Health. (2022) 19:10797. doi: 10.3390/ijerph191710797, PMID: 36078538 PMC9518456

[30] Baran J Weres A Czenczek-Lewandowska E Wyszyńska J Łuszczki E Dereń K. Blood lipid profile and body composition in a pediatric population with different levels of physical activity. Lipids Health Dis. (2018) 17:171. doi: 10.1186/s12944-018-0817-2, PMID: 30045723 PMC6060495

[31] Ørntoft C Larsen MN Madsen M Sandager L Lundager I Møller A. Physical fitness and body composition in 10–12-year-old Danish children in relation to leisure-time club-based sporting activities. Biomed Res Int. (2018) 2018:1–8. doi: 10.1155/2018/9807569, PMID: 30687761 PMC6327252

[32] Kazemi S Sani MT Bagherpour T. Comparison of body composition of active and inactive female students. IJBPAS. (2015) 4:639–44.

[33] Kvintova J Sigmund M. Physical activity, body composition and health assessment in current female university students with active and inactive lifestyles. J Phys Educ Sport. (2016) 16:627–32. doi: 10.7752/jpes.2016.s1100

[34] Mateo-Orcajada A González-Gálvez N Abenza-Cano L Vaquero-Cristóbal R. Differences in physical fitness and body composition between active and sedentary adolescents: a systematic review and Meta-analysis. J Youth Adolesc. (2022) 51:177–92. doi: 10.1007/s10964-021-01552-7, PMID: 35031910

[35] Placek K Aniśko B Wójcik M. Application of balneological treatment in the treatment of obesity in children. Acta Biol. (2023) 65:340–3. doi: 10.36740/ABAL202305114

